# Variability among Animals and Incubation Protocols for Ruminant In Situ Degradation Studies with Tropical Feeds

**DOI:** 10.3390/ani12151901

**Published:** 2022-07-26

**Authors:** Amanda de Souza Assunção, Tadeu Eder da Silva, Daiana Quirino, Marcia de Oliveira Franco, Edenio Detmann

**Affiliations:** 1 Department of Animal Science, Universidade Federal de Viçosa, Viçosa 36570-900, MG, Brazil; amanda.assuncao@ufv.br (A.d.S.A.); daiana.f.quirino@gmail.com (D.Q.); 2 Department of Animal and Dairy Sciences, University of Wisconsin, 1675 Observatory Drive 266, Animal Sciences Building, Madison, WI 53706-1205, USA; tdasilva2@wisc.edu; 3 Natural Resources Institute Finland (Luke), FI-31600 Jokioinen, Finland; marcia.franco@luke.fi; 4 Department of Animal Nutrition and Management, Swedish University of Agricultural Sciences, 750 07 Uppsala, Sweden

**Keywords:** degradation rate, non-linear mixed models, rumen dynamics

## Abstract

**Simple Summary:**

The knowledge on the nutritive value of feeds is essential to feed animals with adequate diets and to optimize production with minimal environment impact. In situ degradation is an important tool for nutritionists because it is a reliable, cheap, and fast way to assess information on feed digestion in ruminants. However, the lack of standards procedures for in situ trials with cattle in the tropics may compromise the reliability of information obtained from those studies. Thus, we aimed to generate useful information for animal scientists on how to perform that kind of study using adequate and minimal resources yet keeping accuracy to interpret feed characteristics. Our findings indicated an important variation among animals on the estimates of the rumen degradation rate of feeds, and taking into account that variation can allow for a more adequate comparison among feeds. On the other hand, we also found that an in situ trial cannot be performed using fewer than three animals, otherwise the risk of obtained biased and imprecise information increases. Minimum sets of incubation times were defined and evaluated. They can be used to decrease the costs and the labor when tropical feeds are evaluated through in situ trials with cattle.

**Abstract:**

Our objectives were to evaluate the variability among animals regarding to the degradation rate of the potentially degradable fraction of dry matter, crude protein, and neutral detergent fiber, as well as to establish the minimum number of animals and provide a standardized design of sampling times for in situ ruminal degradation assays of tropical feeds with cattle. Seven feeds were evaluated, four concentrates and three forages. The incubations were performed using five rumen-cannulated Nellore heifers (328 ± 9.8 kg of body weight). The complete sets of incubation sampling times encompassed 16 time points for forage samples (0–240 h) and 13 time points for concentrate samples (0–144 h). The profiles were adjusted using both fixed and mixed model approaches. When the variation among animals on the degradation rate was considered using the mixed model approach, the precision of the adjusted degradation profiles was increased. Moreover, the utilization of a low number of animals increases the probability to obtain biased estimates of degradation rate and increased random variances. A minimum of three animals is recommended for in situ trials with cattle. Minimum designs of sampling times regarding number and position of incubation times were proposed, discussed, and recommended to assess the dynamics of tropical feed degradation.

## 1. Introduction

Digestibility is the most important characteristic that defines the nutritive value of feeds [1] and its quantification can be performed by in vivo, in vitro, and in situ methods. Among these, the data obtained from in vivo evaluations are considered most reliable and accurate. However, in vivo digestibility assays with cattle are very expensive, as they demand a large number of animals and a large amount of feed [2]. Moreover, this type of assay tends to be time consuming and laborious, due to additional procedures, such as feces collection, orts control and analysis, etc. Such constraints create a demand for faster, cheaper, and less laborious alternatives, such as in situ and in vitro methods [3].

The in vitro methods applied to ruminant animals aim to simulate the rumen physical, chemical, and microbiological conditions [4], and should produce precise digestibility estimates [5]. However, the main disadvantage of the in vitro method is the huge difference in relation to in vivo conditions [6].

On the other hand, in situ methods are widely used to assess ruminal degradation kinetic characteristics (i.e., rate and extent of degradation; [6]). These methods have been considered a reference to evaluate the degradation of a whole feed or its components in different nutritional systems [7,8,9]. In addition, in situ methods have also been considered a more reliable replication of rumen digestion compared to the in vivo environment, as they allow for direct contact of the feed with the rumen environment. However, the literature still raises some conflicting information regarding the utilization of either a single animal or a group of animals as donors, due to the likely interference of some intrinsic animal characteristics on feed degradation, and regarding the optimal set of incubation times (i.e., sampling-time designs) to be used to estimate the kinetic parameters of rumen degradation.

Results obtained in the tropics have demonstrated that there are animal influences on in situ degradation parameters [10], which would indicate that an evaluation of the in situ degradation profile obtained with a single animal may bias the degradation rate estimates. Although diet is considered to have the greatest impact on rumen microbial diversity [11,12], there is an intrinsic effect of the host animal on microbial population structures [13,14]. Such a statement supports the use of a group of animals rather than a single one to assess a likely rumen degradation profile using in situ methods. Despite this, there is no consensus in literature on the number of animals needed to perform in situ degradation assays (e.g., one animal, [15]; two animals [8]; three animals [16,17,18]; four animals [19]).

Moreover, in the literature, there is no agreement on the set of incubation times that should be adopted for in situ procedures (e.g., [6,8,18,19,20,21]). However, it is known that the number and order of different incubation times can affect the estimation of the model parameters in a degradation profile [6]. Even though there are suggestions regarding the number and order of incubation times in non-tropical regions (e.g., [6,8]), to the best of our knowledge, no studies have been carried out with such direct objective for feeds produced under tropical conditions. Notably for forages, due to different characteristics of plant cell wall (e.g., lignin content and structure), and the influence of warmer climate on plant growth and digestibility [22,23,24,25], different degradation patterns may be expected in terms of rate and extent of rumen degradation [26], which may require different designs of sampling times for an adequate in situ assay in the tropics.

Thus, our objectives were to evaluate the variability among animals regarding the degradation rate of the insoluble and potentially degradable fraction of dry matter, crude protein, and neutral detergent fiber, and to establish the minimum number of animals as well as to provide a standardized design of sampling times for in situ ruminal degradation assays with cattle using feeds produced under tropical conditions.

## 2. Material and Methods

The experiment was performed at the Animal Science Department of the Universidade Federal Viçosa, Viçosa, Minas Gerais, Brazil. The animal care and handling procedures were approved by Ethics Committee on the Use of Production Animals of the Universidade Federal de Viçosa (protocol number 20/2017).

### 2.1. Samples Characterization

Three forage and four concentrate samples were evaluated as follows: fresh sugarcane (*Saccharum* sp.), corn silage (*Zea mays*), Tifton 85 hay (*Cynodon* sp.), corn grain, soybean hulls, cottonseed meal, and soybean meal. These feeds were chosen based on their representativeness for tropical cattle diets: three of the main forage sources, the main protein concentrates (soybean and cottonseed meals), the main starchy concentrate (corn grain), and the main high-fiber concentrate (soybean hulls).

Fresh sugarcane and silage samples were oven-dried (55 °C). Samples were ground in a knife mill to pass through a 2-mm screen sieve for use in the in situ procedures [8,27,28]. An aliquot of each ground sample was taken and reground to pass through a 1-mm screen sieve for use in chemical analysis.

The dry matter (DM) content was assessed using the Karl Fisher titration [29], whereas nitrogen content was evaluated by the Dumas method using a Vario EL III analyzer (Elementar Equipments, Langenselbol, Germany). Organic matter (OM, method M-001/2), neutral detergent fiber (NDF, method F-012/1), and lignin (acid hydrolysis; method F-005/2) contents were analyzed according to the standard analytical procedures of the Brazilian National Institute of Science and Technology in Animal Science [30]. The NDF contents were evaluated using a heat-stable α-amylase (Temamyl 2X; Novozymes, Araucária, Paraná, Brazil) omitting sodium sulfite, and were expressed including residual ash and protein (Table 1).

### 2.2. Ruminal Incubations Procedures

Five rumen-cannulated Nellore heifers, averaging 328 ± 9.8 kg of body weight, were used. The heifers were housed in individual pens with concrete floor and equipped with individual feeders and drinkers with free access to a complete mineral mixture (90 g/kg of phosphorus) and fresh water.

The basal diet consisted of Tifton 85 hay and a commercial concentrate in the proportion of 80:20 on a DM basis. The concentrate ingredients were ground corn, soybean meal, wheat bran, urea, ammonium sulfate, minerals and sodium bicarbonate. The diet had 120 g of crude protein (CP) per kg DM, which was fed *ad libitum* twice a day at 0600 and 1800 h. The animals were adapted to the experimental diet for 14 d before the incubations [31]. During the experiment, we performed evaluations regarding the rumen fermentation characteristics of the animals. The methods and results are presented as an Supplementary Materials.

Three sequential incubations were performed, each containing a group of feeds, as follows: group 1—sugarcane, corn silage, and Tifton 85 hay; group 2—soybean meal and corn grain; group 3—soybean hulls and cottonseed meal. In each run/group, the samples were incubated in all animals.

The incubation bags were 8 × 15 cm and made of nylon textile with 50 μm of porosity (Sefar Nitex, Sefar, Switzerland). Aliquots of 6.0 g of ground samples (2-mm screen sieve) were added to individually identified nylon bags, maintaining to proportion of 20 mg DM/cm^2^ of surface [32]. The set of incubation times for forage samples was: 0 h, 3 h, 6 h, 9 h, 12 h, 18 h, 24 h, 30 h, 36 h, 48 h, 72 h, 96 h, 120 h, 144 h, 168 h, and 240 h; while for concentrate samples was: 0 h, 3 h, 6 h, 12 h, 18 h, 24 h, 30 h, 36 h, 48 h, 72 h, 96 h, 120 h, and 144 h. The number of bags per incubation time varied in order to obtain enough residue for laboratory analyses. In this sense, for forage samples, we used: one bag from 0 to 48 h, and two bags from 72 h to 240 h. For concentrate samples, we used: one bag for 0 and 3 h, two bags from 6 h to 12 h, three bags for 18 h, four bags from 24 h to 36 h, and five bags from 48 h to 144 h.

The incubation times were arranged in the rumen in a reverse order so that all bags were removed at the same time [32]. Bags used for time 0 h were introduced into the rumen for enough time for hydration (1–2 min). At the end of incubation, bags were removed and washed in running water to remove the excess of residues from outer bag, and then washed in a washing machine for 5 cycles of 1 min each [8,27]. Finally, the bags were then oven-dried (55 °C) and weighed.

The forage incubation residues were analyzed for DM, CP, and NDF contents, whereas the concentrate incubation residues were analyzed for DM and CP contents. We followed the methods described above.

### 2.3. Evaluation of Variability among Animals Regarding Degradation Rates

The evaluation of variability among animals was based on the degradation rate assuming that undegradable and potentially degradable fractions are intrinsic characteristics of the feeds [33].

However, to validate this assumption, we performed an analysis of variance (ANOVA) on the proportion of residues obtained at time 0 h and at the longest incubation times for DM and CP of each feed, and at the longest incubation time for forage NDF. The residue obtained at 0 h was supposed to be a direct approximation of the limit between soluble and insoluble fractions of DM and CP. On the other hand, we assumed that the longest incubation time could be used as an approximation for the limit between potentially degradable and undegradable fractions for all components evaluated here.

The ANOVA were performed independently for each incubation time (0 h and longest time), feed type (forage or concentrate), and evaluated component (DM, CP, or NDF), according to the model:

(1) $$Y_{ij}=\mu +F_{i}+A_{j}+\varepsilon_{ij}$$

where *Y_ij_* is the observation obtained for feed *i* in animal *j*; *µ* is the general constant; *F_i_* is the effect of feed *i* (fixed); *A_j_* is the effect of animal *j* (random), assumed NIID (0, σ^2^_a_); and *ε_ij_* is the random error, assumed NIID (0, σ^2^_ε_).

The ANOVA were performed using the MIXED procedure of SAS 9.4. The significance of the variance component associated with the variation among animals was evaluated using the Wald Z score [34] with α = 0.05. It was anticipated that none of the evaluations performed according to the model (1) indicated variability among animals (*p* > 0.05), corroborating our earlier assumption and supporting the later evaluations that will be described below.

From the graphical evaluation of the incubation residues over time, a first-order exponential model [35] was chosen to describe the DM and CP degradation profiles, and a gamma-2 time-dependent model [36] was chosen to describe NDF degradation. Both models were adapted to a mixed model form including one parameter associated with random variability among animals on the degradation rate of insoluble and potentially degradable fraction, as follows:

(2) $$D_{ij}=A+B\times \left[1-e^{-k\left(\pm u_{i}\right)\times t_{j}}\right]+\varepsilon_{ij}$$

(3) $$R_{ij}=B\times \left[1+\lambda \left(\pm u_{i}\right)\times t\right]\times e^{-\lambda \left(\pm u_{i}\right)\times t_{j}}+U+\varepsilon_{ij}$$

where *D_ij_* is the degraded fraction of DM or CP obtained in animal *i* at incubation time *j* (g/100 g); *A* is the soluble fraction (g/100 g); *B* is the insoluble and potentially degradable fraction (g/100 g); *k* is the fractional rate of degradation of DM or CP (h^−1^); *u*_i_ is the parameter associated with the random effect of animal *i* on *k* or *λ*, which is assumed to have an asymptotic normal distribution, with mean 0 and variance σ^2^_a_; *t_j_* is the incubation time j (h); *R_ij_* is the undegraded residue of NDF obtained in animal *i* at incubation time *j* (g/100 g); *λ* is the time-dependent rate parameter related to NDF degradation (h^−1^); *U* is the undegradable fraction (g/100 g); and *ε_ij_* is the random error, which is assumed to have an asymptotic normal distribution independent of *u*_i_, with mean 0 and variance σ^2^_ε_.

**Figure 1:**
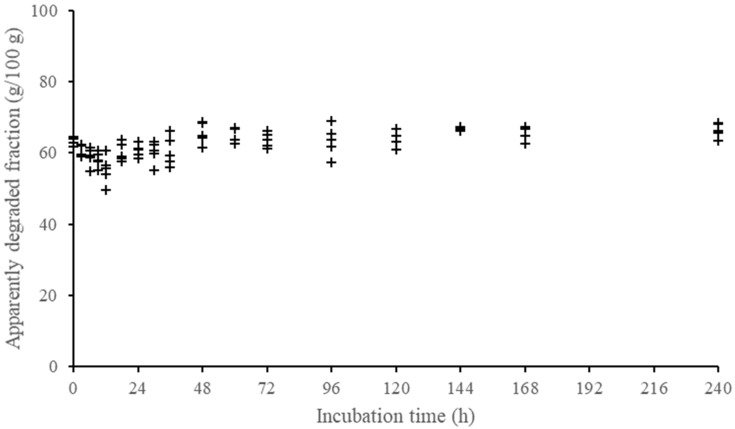
Descriptive pattern of the apparently degraded fraction of crude protein of a forage sample (sugarcane) according to incubation time (each point within incubation time represents one different animal).

We emphasize that concentrates were not evaluated for fiber degradation due to the low contribution of this component in most concentrate feeds and to the difficulty in obtaining incubation residues that could allow an adequate evaluation of NDF degradation. On the other hand, during the experiment, we observed that apparently undegraded forage CP residues showed a biologically unlikely behavior, possibly attributed to the low CP content associated with a high microbial contamination. An example of that pattern is shown in Figure 1. A correction for microbial contamination was performed based on equation proposed by Machado et al. [21]. However, that correction seems to have altered the random variation of data, resulting in systematically negative estimates of the variance component associated with degradation rate. Thus, due to these systematic and illogical results (i.e., negative variances), we decided to omit the evaluation of CP degradation profiles for forage samples.

Initially, models (2) and (3) were adjusted to data omitting the random variation among animals on degradation rate (i.e., fixed model) using the NLIN procedure of SAS 9.4. The adjustments were based on the iterative method of Gauss–Newton. The initial estimates of the different fractions were calculated based on the incubation residues obtained at 0 h and at the maximum incubation time used. The initial estimates for the parameters k and λ were obtained through the grid-search statement of the NLIN procedure.

The solution obtained through the fixed model was used to provide the initial estimates for the adjustment of models (2) and (3) (mixed models) using the NLMIXED procedure of SAS 9.4. The adjustments were performed using the first-order integration method for maximum likelihood of random effects [37] with the *Dual Quasi-Newton* optimization technique. Significance of the variance component associated with the animal effect on degradation rate was evaluated by an asymptotic approximation to *Student t* distribution. Due to the high probability of occurrence of type II error, the significance for this variance component was declared at *p* < 0.10. In addition, the quality of the fixed and mixed model adjustments to the data was evaluated through the Akaike information criterion (AIC) [38].

After the adjustments, the degradation rates (*k* and *λ*) obtained by fixed and random models were compared by fitting a simple linear regression model using the following hypotheses:

(4) $$H_{0}{:\beta }_{0}={0\;\mathrm{and}\;H}_{a}{:\beta }_{0}\neq 0$$

(5) $$H_{0}{:\beta }_{1}={1\;\mathrm{and}\;H}_{a}{:\beta }_{1}\neq 1$$

The degradation rates obtained by fixed and random model adjustment were considered equal when both null hypotheses were not rejected (*p* > 0.05).

### 2.4. Evaluation of the Minimum Number of Animals for In Situ Degradation Essay

To evaluate the influence of the number of animals used to adjusted a degradation profile, mainly regarding the pattern of degradation rates (*k* and *λ*), the Equations (2) and (3) were adjusted on all possible combinations of the five animals in groups of two (ten combinations), three (ten combinations) and four (five combinations). All the adjustments were performed taking into account the random variation among animals on the degradation rate using the NLMIXED procedure of SAS 9.4, as previously described.

The degradation profiles adjusted using different number of animals were compared in terms of the values of variance among animals associated with degradation rate and residual variance, keeping as the reference the values obtained using all animals (n = 5).

### 2.5. Establishment of a Minimum Design of Sampling Times for Incubations

The evaluations to establish a minimum design of sampling times for in situ incubations were initially performed for the DM degradation profiles of all feeds, according to the following procedures. First, the degradation profiles were adjusted considering all the incubation times (16 times for forages and 13 times for concentrates). From this adjustment, asymptotic confidence intervals (1 – α = 0.95) were estimated for the parameters A, B, and k (Equation (2)). We opted for the fixed model adjustment and used the NLIN procedure of SAS 9.4, as previously described. Our option is supported by the narrower confidence intervals using a fixed model. By assuming this, we were able to achieve a greater sensitivity to detect distortions in the degradation profiles caused by the omission of one or more incubation times.

Our first objective was to establish the optimal value for the final incubation time. Decreasing the final incubation time will decrease the time spent with the whole incubation procedure. In this way, the times were gradually withdrawn, one by one, going back in relation to the last time used in this study (240 h for forages and 144 h for concentrates) and a new adjustment was performed after each time withdrawal. This procedure was repeated until at least one of the parameters estimates (A, B, or k) obtained with the reduced design has been found outside its respective asymptotic confidence interval estimated with the total set of incubation times. Thus, this point was kept in the design, as its removal would cause a significant bias in the estimates of one or more parameter.

After defining the incubation end point, intermediate time points were removed from the profile aiming to increase the time interval among incubation times. An incubation time was considered unnecessary when its removal did not compromise the estimates of the parameters A, B, and k (Equation (3)) according to asymptotic confidence intervals obtained with the total set of incubation times. The removal of points in middle and final thirds of the incubation profile was prioritized, since it is known that there is a need for a greater number of times in the initial third than during other periods of fermentation to offset the greater variation that occurs during this period of rapid degradation [6]

After the establishment of the minimum design of sampling times to evaluate the concentrate DM degradation, the evaluation of the minimum design for the degradation of the concentrate CP was carried out. In this case, we assumed that the first logical step would be to verify the suitability of the design defined for DM to assess the CP degradation. Then, the degradation profiles were adjusted considering the total set of incubation times to obtain the asymptotic confidence intervals (1 – α = 0.95) for the degradation parameters (Equation (2)). Afterwards, the profiles were adjusted the model again considering the minimum design of sampling times obtained for DM degradation. If the parameter estimates (A, B, and k) were found within the asymptotic confidence boundaries, our conclusion would be that the minimum design for DM degradation could also be used for the assessment of CP degradation.

A similar procedure was adopted for the evaluation of minimum design for forage NDF degradation. The adjustments were initially made considering the entire set of incubation times to obtain the respective asymptotic confidence intervals (1 – α = 0.95) for the parameters B, U, and λ (Equation (3)). Afterwards, the profiles were adjusted again considering the minimum incubation designs obtained for forage DM degradation. If the estimates of B, U, and λ were found between asymptotic confidence boundaries, we would conclude that the minimal sampling time design for DM is also adequate to assess NDF degradation. However, we anticipate that such adequacy was not observed (*p* < 0.05), mainly because the overestimation of undegradable NDF caused by short incubation periods, which is supported by results presented by other authors in the tropics [39,40].

To overcome that situation, an additional incubation time was included in the minimum DM design to evaluate the NDF degradation. All time points above the maximum time used for DM degradation were included one at a time until the estimates of the degradation parameters were found within the boundaries of their respective asymptotic confidence intervals.

When all minimum designs of sampling times were established, their residual variances were compared with those obtained with the total set of incubation points by the Snedecor-Fisher test (α = 0.05).

## 3. Results and Discussion

### 3.1. Comparison of Adjustments Using Fixed or Mixed Models Approach

There was no variation among animals (*p* ≥ 0.20) regarding the undegraded residues obtained at time 0 and at the longest incubation times used in this study (Table 2). Such evaluation was performed under the assumption that those incubations times represent adequate approaches for the borderline between soluble and insoluble fractions (i.e., time 0) and between potentially degradable and undegradable fractions (i.e., longest incubation times). The pattern here obtained corroborates the hypothesis that the fractions of the different feed components are intrinsic characteristics of the feeds themselves [33]. This pattern also supports our previous decision of centering our evaluations regarding random variation on degradation rates.

**Figure 2:**
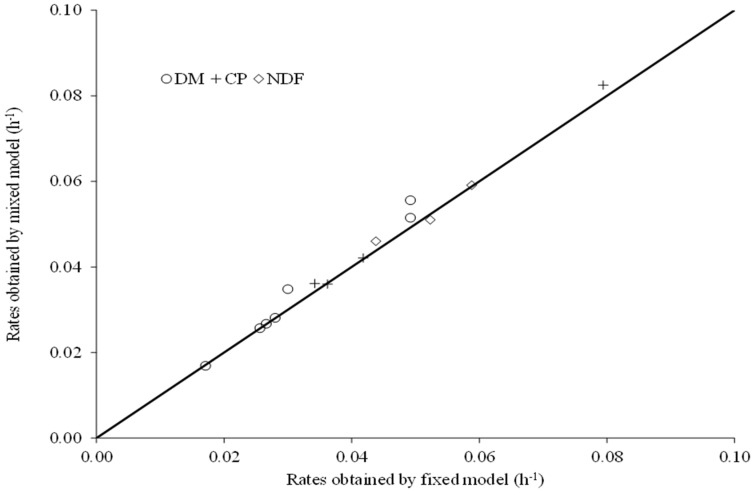
Relationship between degradation rates (DM, dry matter; CP, crude protein; NDF, neutral detergent fiber) of different feeds estimated by the fixed or mixed nonlinear modelling approaches [Ŷ = −0.00007 – 1.037 × X; r^2^ = 0.98; s_XY_ = 0.002; *p* value (β_0_ = 0): 0,964; *p* value (β_1_ = 1): 0.342; the continuous line represents the relation of equality].

For all the degradation profiles studied, except for CP from soybean hulls, we obtained adjustments with estimates of variance among animals on the degradation rate numerically greater than zero (Table 3). However, in spite of the peculiarities associated with each modelling approach, there were no differences (*p* ≥ 0.34) in the degradation rates estimates obtained by the fixed or mixed models (Figure 2). This pattern seems to be logical, as the mathematical expectations of the parameters are the same for both approaches [41,42].

In this sense, the main difference between fixed and mixed model approaches relies on the precision of the adjusted degradation profiles. For the 13 profiles with numerically positive variance among animals, only one provided a variance component different from zero (*p* < 0.08, sugarcane NDF, Table 3). At first glance, such a pattern seems to indicate that there is no variation among animals regarding degradation rate. However, we must emphasize an intrinsic limitation of the method we used here to point out the significance of the variance components. Actually, we applied an asymptotic approach to the Student’s t distribution, whose statistical diagnostics could be compromised by the restricted sample size and, consequently, by the degrees of freedom in the test denominator (d.f. = 4).

However, the variation among animals can be indirectly inferred from the size of residual variance (i.e., error), which quantifies the variation caused by any source of variation that is not covered by the model structure. When we considered variation among animals on degradation rate, we observed decreased values of residual variance for all adjusted degradation profiles (Table 3). This specific pattern accrues from the fixed model does not consider animals as a known source of variation and includes that along with other unknown variabilities in the residual variance.

Considering that degradation rates influence the values of degraded/undegraded residues over the different incubation times (excepting at time 0 and infinite) and also that animal can influence the estimates of this parameter, it should be expected that such variability would be considered to come from any unknown source of variation in a fixed model approach. Conversely, when a mixed model approach is utilized, variation among animals becomes a known source of variation, which implies a decrease in residual variation as well as an increase in precision of the adjusted degradation profile. In this case, a decreased residual variance points to the animal influences on the degraded/undegraded residues along time. According to our results (Table 3), the variation among animals corresponded to 11.9–46.7% (averaging 24.3%) of the total random variability of the degradation profiles. Vanzant et al. [27], after compiling data from literature, pointed out that variation among animals accounted for 40% of the total random variance on the values of the degraded fraction, which agrees with our results herein.

The degradation rate is an intrinsic function of the substrate and the digestion environment [6] and its expression depends on the intensity of microbial activity on the substrate [28]. The term “rumen environment” encompasses all major factors that can affect the activity of microbial enzyme systems on substrates, such as pH, minerals, nitrogen compounds (i.e., ammonia and peptides), branched-chain fatty acids, etc. [43].

The feed itself is a potential supplier of substrates for microbial growth, as well as influences the physical-chemical characteristics of the rumen. Consequently, the rumen environment conditions are interrelated with feed characteristics [33]. However, in this work, both basal diet and incubated feeds were constant among animals. Thus, the variability in degradation rate among animals was likely caused by the animals themselves. Many rumen environment aspects are directly influenced by the animal (e.g., buffer release, temperature maintenance, nitrogen recycling, etc., [44]). Moreover, there are intrinsic effects of host animal on the structure of rumen microbial populations [13,14], which may explain the variability among animals regarding degradation rate. It must be noticed that the animals used in our study were homogeneous and variations attributed to effects of body size, genetic group, and physiological maturity are not expected to influence the results.

**Figure 3:**
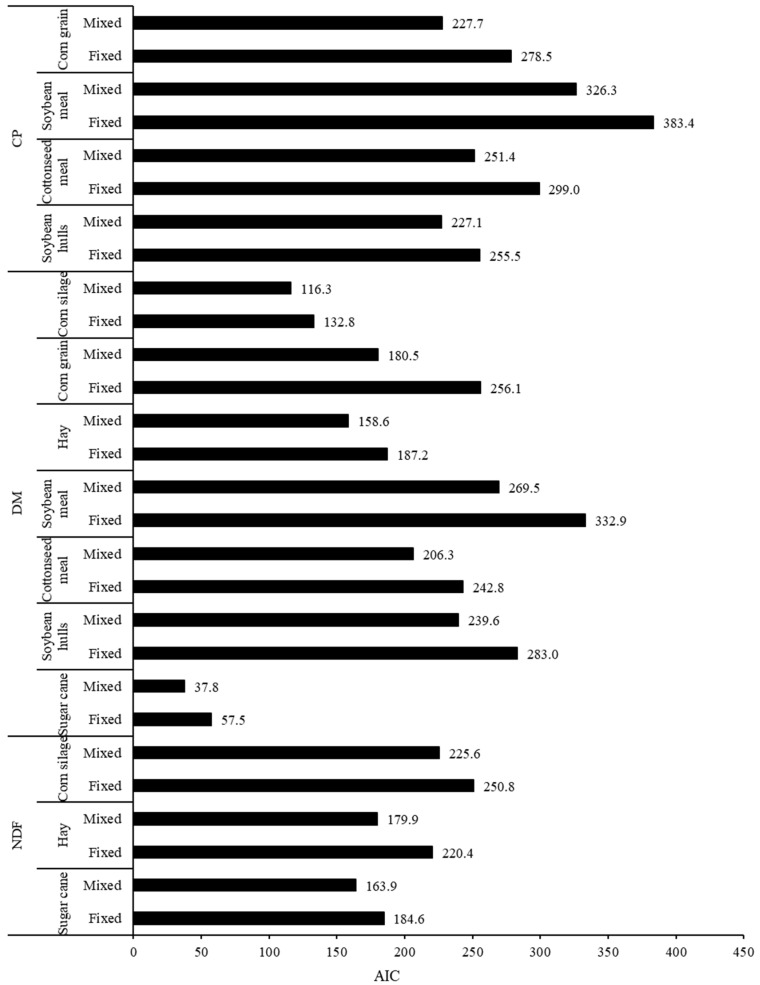
Pattern of the Akaike information criterion (AIC) for the adjusted degradation of dry matter (DM), crude protein (CP), and neutral detergent fiber (NDF) for different feeds by using the fixed or mixed non-linear modelling approaches.

Conversely, the decrease in residual variance associated with the mixed model approach could be partially attributed to the consideration of one additional parameter in the models (parameter μ, Equations (2) and (3)). Normally, for any model, a greater number of degrees of freedom associated with the known variation source leads to a numerically decreased residual variance, but it does not necessarily mean that a better adjustment of model to the data has been achieved. Thus, a more comprehensive evaluation must be performed considering simultaneously the decrease in residual variation and the number of degrees of freedom spent to achieve that decrease (which equals the number of new parameters added to the model) [38]. In this sense, the inclusion of parameter μ in the mixed model approach provided consistent improvements in the adjustment to the data, as the AIC values were lower than those obtained in the fixed model approach for all evaluations here performed (Figure 3).

Having said that, considering the lack of influence on the degradation rate estimates (Figure 2), the obvious advantage for using a mixed model approach relies on residual variance reduction (Table 3). In this sense, any effective practical benefit from using non-linear mixed models would be observed if the incubation procedures were linked to an experimental design in which the main objectives relies on statistical comparison between feeds or diets. In those cases, the residual variance is a fundamental element for the comparison procedures, which would be more precise as the random error is reduced. Nevertheless, despite this gain in precision, when the assays only aim to estimate the feed degradation parameters, without any statistical comparisons among feeds, both fixed and mixed approaches could be used due their similar accuracy (Figure 2). However, a fixed model approach would provide a huge operational advantage, as it is less complex and converges faster through iterative algorithms, which are also less sensitive to the use of non-optimal starting values.

### 3.2. Evaluation of the Number of Animals Used in the Incubation Procedures

**Figure 4:**
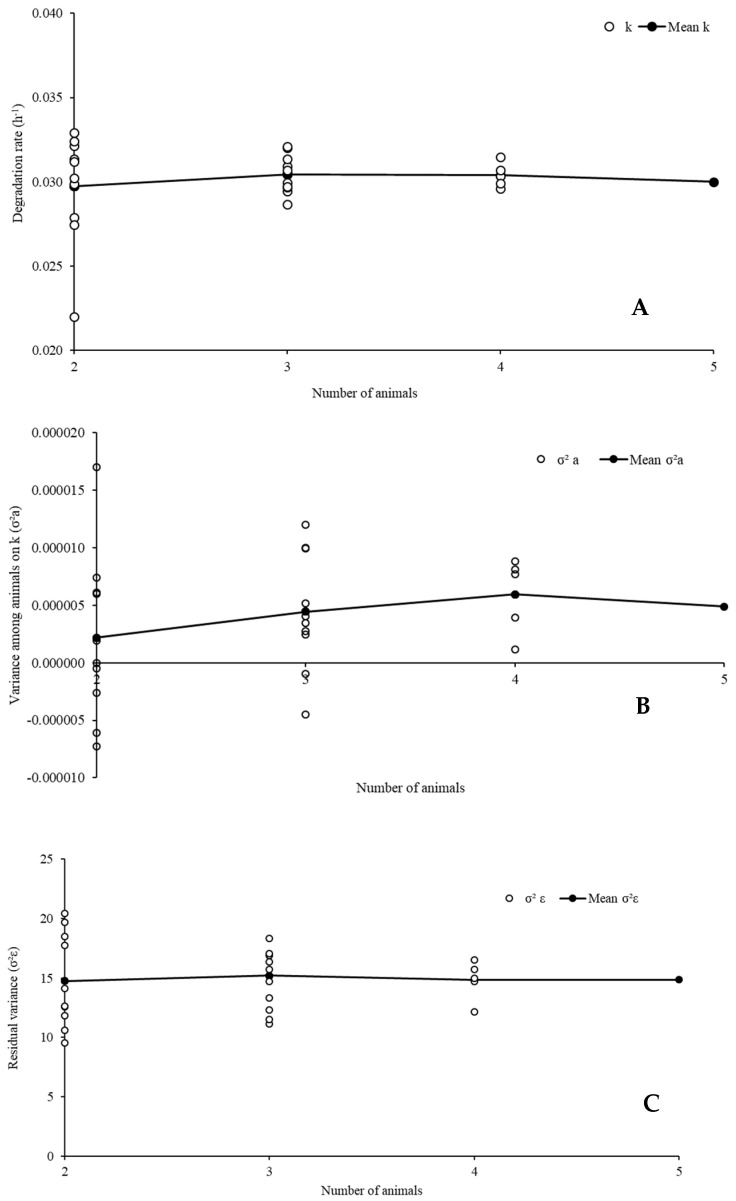
Variability of the estimates of degradation rate of the potentially degradable insoluble fraction of the dry matter (k), (**A**), random variance among animals on k (σ^2^_a_), (**B**), and residual variance (σ^2^_ε_), (**C**) for the dry matter degradation profile of cottonseed meal according to the number of animals used in the in situ trial.

The patterns of the adjusted degradation profiles considering different numbers of animals were similar for all evaluated feeds and components. Thus, only examples of the results regarding DM degradation evaluation of cottonseed meal were shown in graph (Figure 4).

The variation in the number of animals had no drastic effect on the average estimates of degradation rate, variance among animals, and residual variance (Figure 4). Such a pattern contradicts the arguments presented by other authors [16,27,45], who pointed out an increased precision as the number of animals used for incubation procedures increased. However, in spite of the average pattern, we observed that, as the number of animals decreased, the dispersion of the evaluated characteristics increased (Figure 4). Such a pattern was our first evidence that the use of a single animal can lead to widely dispersed estimates of degradation parameters compared to the average expected value for a feed. In fact, we detected a great variation between animals regarding ruminal characteristics (Supplementary Materials), which reinforces our previous argument.

The random effect of animals may manifest positively or negatively on the degradation rate. The major concern here relies on the fact that it can only be perceived after the study has been performed and if, and only if, more than one animal is used. This latter affirmative is important because, as a random effect, the variability between animals can only be perceived and measured when true replicates are used in the study. Otherwise, the variation is not observable.

The dispersion of the characteristics evaluated herein (Figure 4) indicates that using a small group of animals may compromise both accuracy and precision in degradation assays. Such a constraint occurs due to the increased risk of obtaining estimates that are far from what would be expected with a larger number of animals. If only two animals are used, the risk of a biased animal influence from extreme random effects, both positive and negative, increases. This can even lead to the estimation of negative variances associated with inter-animal variability on degradation rate (Figure 4), which is a mathematical inconsistency, and demonstrates convergence glitches if mixed models need to be used.

**Figure 5:**
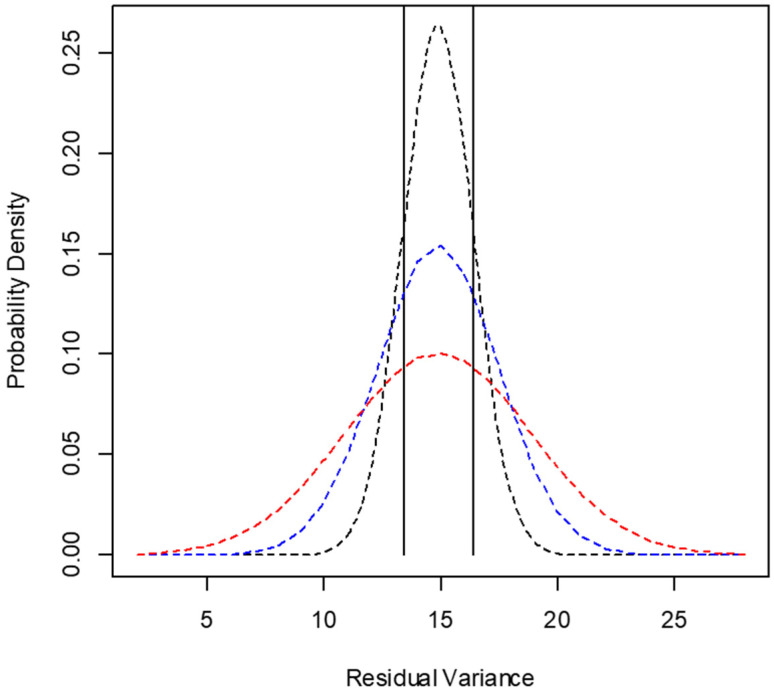
Example of probability distribution according to the central limit theorem for the residual variance of cottonseed meal DM degradation according to the number of animals used in the in situ trial [dashed lines: black = 4 animals; blue = 3 animals; red = 2 animals; the vertical lines delimit the region for *p* (0.9 × σ^2^_Ԑ_ < σ^2^_Ԑ_ < 1.1 × σ^2^_Ԑ_)].

Considering the low influence of the number of animals on the average estimates (Figure 4), the dimension of the risk for obtaining extreme values of degradation rate and for variance components was estimated based on the central limit theorem [46], where it is assumed that samples of parameter estimates taken from non-normal populations tend to present a normal distribution. The mean value obtained with five animals was assumed as the population mean, and the distributions were adjusted according to the variances among estimates obtained with two, three, or four animals. For each evaluation, the probability of obtaining values centered on population mean in ±10% was estimated (Figure 5, Table 4). As we observed some negatives estimates of variance associated with animals’ effect on degradation rate (Figure 4), we also estimated the occurrence probability for this pattern when using two, three, or four animals in the incubation procedures (Table 4).

**Figure 6:**
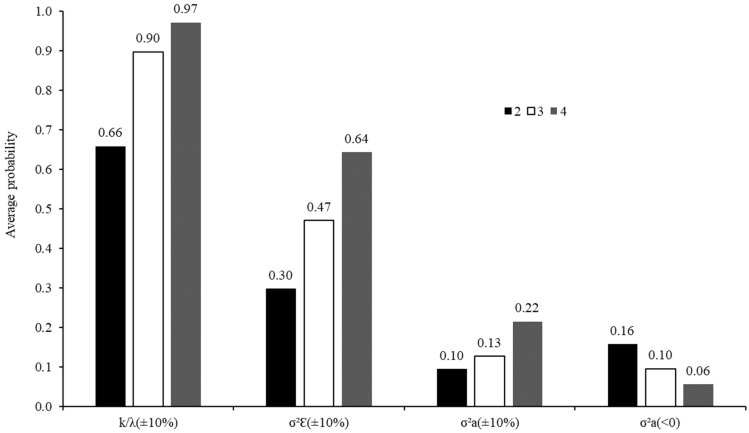
Average probabilities for obtaining estimates of degradation rate [k/λ (±10%)], error variance [σ^2^_Ԑ_ (±10%)], and variance among animals on degradation rate [σ^2^_a_ (±10%)] lower or higher than 10% in relation to those obtained using five animals, and average probabilities for obtaining negative estimates of variance among animals on degradation rate according to the number of animals used in the in situ trial.

The corn grain presented atypical behavior regarding the CP degradation, with an extremely higher dispersion compared to the other adjusted profiles (Table 4). Therefore, this information has been omitted from the other discussions in this topic. In general, as the number of animals increased, both risks of bias in the estimates and loss of precision were decreased (Table 4; Figure 6). It likely represents a dilution effect of extreme random effects in a larger group of animals, causing random effects that are less dispersed and closer to their population mean (assumed to be zero; Equations (2) and (3)).

In particular, the probability of obtaining degradation rate estimates similar to that obtained with five animals increased as the number of animals increased. However, there was no clear differentiation between using three or four animals, whose probabilities were, on average, very close to each other and equal to or greater than 0.9 (Figure 6). Thus, in trials where the objective is to estimate the degradation rate, the utilization of at least three animals would incur in a lower risk of obtained extreme estimates that are far from the expected values. This recommendation corroborates the ones made by Mehrez and Ørskov [16] and Åkerlind et al. [17], whose studies are all based on using three animals. However, it became understood that the recommendations of Tomich and Sampaio (ref. [15], one animal) and NRC (ref. [8], two animals) are not suitable for evaluating tropical feeds.

The recommendations presented here, even though directed to in situ degradation assays with a single incubation run, may be extended to assays with different arrangements or designs. Due to the need for simultaneous evaluation of a large number of feeds, some authors have used more complex experimental designs, mainly Latin squares [18,21,39], in which feed samples are divided in groups, and those are alternately incubated in different animals for consecutive periods. In these trials, there is an experimental control of variability among animals, but there is also the risk of outlier random effects by using a low number of animals. Thus, also for this type of assay, the recommendation to use at least three animals seems relevant.

However, regarding the pattern observed for degradation rate, the average probabilities related to the variance components (Figure 6) did not allow to establish a minimum number of animals for degradation trials. For the three components analyzed, a linear pattern was observed. As the number of animals increased, the probability of obtaining variance components closer to that obtained with five animals also increased, whereas the probability of obtaining negative components of variance for animal effects on the degradation rate decreased. Thus, the results obtained here do not seem to be totally conclusive concerning the ideal number of animals, mainly regarding trials where comparisons among feeds would be the main objective. However, although not conclusive, the results allow us to infer that an increased number of animals decrease the probability of obtaining extreme estimates of variances, which could compromise the comparison procedures.

### 3.3. Definition of Minimum Scheme of Incubation Times

Nine incubation time points were defined as the minimum design to evaluate the DM degradation profiles (Table 5). We highlight that within both forages and concentrates, there was a convergence in the incubation times, which evidences the adequacy and robustness of our proposal protocols. The minimum designs produced similar estimates for DM degradation parameters (*p* > 0.05) when compared to the whole set of incubation times, while not affecting the residual variance estimates (*p* ≥ 0.11, Table 6). This shows similarity in both accuracy and precision.

Using a minimum design of sampling times based on nine incubation times converges to the recommendation of Mertens [6], who affirmed that the estimation of degradation profiles could be assured using at least three incubation times for each parameter in the model. The models used to define the minimum incubation schemes consisted of only three parameters (B, k or λ, and A or I). Thus, based on those statements, if the applied model is composed for more than three parameters, the minimum designs suggested here could be altered by adding more incubation times.

The minimum design of sampling times defined for DM were equally effective in evaluating the CP degradation pattern in concentrate feeds (Table 6). Thus, a single set of incubation times may be used to evaluate DM and CP degradation in concentrates.

For forage NDF degradation, when using the minimum design of sampling times established for DM degradation, the estimates of parameters B and U were biased (*p* < 0.05) when compared to the values obtained with whole set of incubation times (data not shown). This pattern was caused by a systematic overestimation of undegradable fraction of NDF. Thus, we chose to keep the minimum design for DM but adding an additional incubation time to achieve unbiased estimates of both potentially degradable and undegradable NDF fractions. Then, we decided to evaluate among the longer times the one that could be used for all the forages evaluated in this study, allowing to produce a general recommendation. This additional time was set at 240 h. Its inclusion in the minimum design guaranteed the similarity of the estimates of the parameters B, U, and λ (*p* > 0.05), and the residual variance (*p* ≥ 0.15) when compared to the values obtained with the complete set of incubation times (Table 6).

The main difference among the minimum sampling-time designs proposed in this study and those highlighted in the literature for non-tropical conditions relies on the value of the longest incubation time. In our work, 96, 120, and 240 h are recommended for studying the degradation of concentrate DM/CP, forage DM, and forage NDF, respectively (Table 5).

The NRC [8] standard procedures for CP degradation studies are based on maximum incubation times of 48 h and 72 h for concentrates and forages, respectively. Moreover, the designs of sampling times proposed by Mertens [6] for rapidly and slowly digesting feed components are based on maximum incubation times of 64 h and 96 h, respectively. If applied to the feeds here evaluated, both proposals would produce biased degradation profiles.

Taking proper information at the end of the degradation process is critical to accurately estimate the extension of degradation [6]. In this sense, using very short times may lead to the overestimation of undegradable fraction. Feeds produced under tropical and non-tropical conditions are likely to differ in terms of ruminal degradation dynamics [28]. This difference seems to support the need for longer incubations periods for the feeds produced in the tropics.

## 4. Conclusions

Taking the animal random variation into account does not influence the in situ estimates of degradation rate, but improves the precision of the adjusted models. A minimum of three animals is recommended for in situ ruminal degradation studies with cattle. Minimum designs of sampling times for in situ trails with cattle were proposed, discussed, and recommended to assess the dynamics of tropical feeds degradation.

## Figures and Tables

**Table 1:** Chemical composition of the feeds.

Feeds ^1^	DM	OM	CP	NDF	Lignin
	g/kg	g/kg DM			
Sugarcane	303	971	35.9	515	52.1
Corn silage	287	915	112	373	33.1
Tifton hay	878	929	114	771	54.2
Soybean meal	890	940	543	165	2.6
Corn grain	883	988	105	105	2.1
Soybean hulls	886	955	197	652	19.1
Cottonseed meal	940	941	465	364	89.3

^1^ DM, dry matter; OM, organic matter; CP, crude protein; NDF, neutral detergent fiber.

**Table 2:** Evaluation of the variability among animals regarding the non-degraded fractions obtained in the initial (0 h) and final (144 h for concentrates and 240 h for forage) incubation times according to the type of feed and evaluated component.

Feed Type	Component ^1^	Time (h)	*p* Value ^2^
Forage	DM	0	0.888
		240	0.209
	NDF	240	0.308
Concentrates	DM	0	0.818
		144	0.836
	CP	0	0.868
		144	0.605

^1^ DM, dry matter; NDF, neutral detergent fiber; CP, crude protein, ^2^ *p* value associated to the significance of component of variance among animals (Equation (1)) obtained by the Wald Z-test.

**Table 3:** Characteristics of estimation process of the parameters associated with degradation rate (k ou λ, h^−1^) in different feeds and feed components using fixed or mixed modelling approaches.

	Fixed Model ^1^				Mixed Model ^2^				
Feed	k/λ	ASE	σ^2^_Ԑ_	k/λ	ASE	σ^2^_a_	*p* value	σ^2^_Ԑ_	Δ(%)
Dry Matter									
Sugarcane	0.0171	0.00093	1.90	0.0169	0.00168	3.57 × 10^−6^	0.513	1.49	21.6
Corn silage	0.0256	0.00123	4.61	0.0257	0.00146	4.61 × 10^−6^	0.235	3.75	18.7
Tifton hay	0.0266	0.00126	8.74	0.0264	0.00170	9.55 × 10^−6^	0.154	6.17	29.4
Soybean meal	0.0492	0.00440	48.75	0.0511	0.00607	1.30 × 10^−4^	0.313	34.50	29.2
Corn grain	0.0492	0.00272	19.74	0.0482	0.00392	7.20 × 10^−5^	0.219	10.53	46.7
Soybean hulls	0.0280	0.00198	27.11	0.0281	0.00204	6.13 × 10^−6^	0.214	23.18	14.5
Cottonseed meal	0.0300	0.00230	16.88	0.0303	0.00251	6.87 × 10^−6^	0.330	14.87	11.9
Crude Protein									
Soybean meal	0.0418	0.00392	88.28	0.0421	0.00405	2.90 × 10^−5^	0.408	73.59	16.6
Corn grain	0.0342	0.00335	25.71	0.0361	0.00447	4.60 × 10^−5^	0.355	19.78	23.1
Soybean hulls	0.0362	0.00402	19.61	0.0360	0.00322	0	---	19.61	---
Cottonseed meal	0.0794	0.00537	32.73	0.0825	0.00778	1.32 × 10^−4^	0.399	27.13	17.1
Neutral Detergent Fiber									
Sugarcane	0.0438	0.00161	8.47	0.0438	0.00170	1.40 × 10^−5^	0.078	6.57	22.4
Corn silage	0.0523	0.00186	18.46	0.0524	0.00264	2.90 × 10^−5^	0.248	13.57	26.5
Tifton hay	0.0588	0.00200	12.91	0.0591	0.00320	5.20 × 10^−5^	0.143	7.93	38.6

^1^ ASE, asymptotic standard error of k or λ; σ^2^_Ԑ_, residual variance. ^2^ ASE, asymptotic standard error of k or λ; σ^2^_a_, variance component associated with variation among animals on k or λ; *p* value, significance associated with H_0_: σ^2^_a_ = 0; σ^2^_Ԑ_, residual variance; Δ(%), contribution of the variability among animals on the residual variance based on the fixed model adjustment. This value was calculated as the percentage of decrease in σ^2^_Ԑ_ when the mixed model approach was applied instead the fixed model.

**Table 4:** Probabilities to obtain estimates of degradation rate [k/λ (±10%)], error variance [σ^2^_Ԑ_ (±10%)], and variance among animals on degradation rate [σ^2^_a_ (±10%)] lower or higher than 10% in relation to those obtained using five animals, and probabilities to obtain negative estimates of variance among animals on degradation rate according to the number of animals used in the in situ trial.

	σ^2^_Ԑ_ (±10%)			σ^2^_a_ (±10%)			σ^2^_a_ (±10%)			σ^2^_a_ (<0)		
Feed	2	3	4	2	3	4	2	3	4	2	3	4
Dry Matter												
Sugarcane	0.82	0.94	>0.99	0.47	0.74	0.86	0.14	0.16	0.21	0.04	0.02	<0.01
Corn silage	0.53	0.98	>0.99	0.55	0.74	0.93	0.08	0.09	0.13	0.17	0.13	0.06
Tifton hay	0.79	0.94	0.99	0.27	0.44	0.63	0.10	0.12	0.16	0.10	0.07	0.02
Soybean meal	0.45	0.67	0.83	0.16	0.26	0.39	0.09	0.13	0.18	0.13	0.05	0.01
Corn grain	0.51	0.70	0.92	0.18	0.27	0.39	0.13	0.17	0.28	0.05	0.02	<0.01
Soybean hulls	0.77	0.94	>0.99	0.37	0.50	0.70	0.08	0.11	0.16	0.17	0.08	0.02
Cottonseed meal	0.64	0.99	>0.99	0.29	0.44	0.67	0.05	0.08	0.12	0.25	0.17	0.07
Crude Protein												
Soybean meal	0.62	0.77	0.94	0.24	0.39	0.56	0.08	0.13	0.25	0.16	0.05	<0.01
Corn grain	0.14	0.13	0.16	0.22	0.25	0.30	0.00	0.00	0.00	0.49	0.49	0.50
Cottonseed meal	0.33	0.99	>0.99	0.22	0.34	0.50	0.10	0.18	0.53	0.10	0.01	<0.01
Neutral Detergent Fiber												
Sugarcane	0.89	0.99	>0.99	0.26	0.66	0.83	0.08	0.10	0.13	0.14	0.10	0.05
Corn silage	0.79	0.94	>0.99	0.30	0.50	0.71	0.10	0.13	0.18	0.12	0.06	0.01
Tifton hay	0.77	0.92	>0.99	0.26	0.39	0.57	0.11	0.13	0.26	0.08	0.05	<0.01

**Table 5:** Description of selected incubation times to establish minimum designs of sampling times for in situ degradation studies of forage and concentrate dry matter.

Incubation Times (h) ^1^																
Feed	0	3	6	9	12	18	24	30	36	48	72	96	120	144	168	240
Forages																
Sugarcane	×	×	×	-	×	-	×	×	-	×	×	-	×	-	-	-
Corn silage	×	×	×	-	×	-	×	×	-	×	×	-	×	-	-	-
Tifton hay	×	×	×	-	×	-	×	×	-	×	×	-	×	-	-	-
Concentrates																
Soybean meal	×	×	×	n	×	-	×	×	-	×	×	×	-	-	n	n
Corn grain	×	×	×	n	×	-	×	×	-	×	×	×	-	-	n	n
Soybean hulls	×	×	×	n	×	-	×	×	-	×	×	×	-	-	n	n
Cottonseed meal	×	×	×	n	×	-	×	×	-	×	×	×	-	-	n	n

^1^ (×), times selected; (-), omitted times. (n), not evaluated.

**Table 6:** Estimates of the degradation parameters of the dry matter, crude protein, and neutral detergent fiber of the different feeds considering the complete set of incubation times and the minimum designs of sampling times.

	Complet Time Set					Minimal Design ^1^					
Feed	A	B	U	k/λ	σ^2^_Ԑ_	A	B	U	k/λ	σ^2^_Ԑ_	*p* Value ^2^
Dry Matter											
Sugarcane	48.5	26.1	-	0.0171	1.90	48.7	26.9	-	0,0159	2.48	0.151
Corn silage	47.3	39.2	-	0.0256	4.61	47.7	38.4	-	0.0258	5.60	0.224
Tifton hay	21.7	54.2	-	0.0266	8.74	22.4	54.2	-	0.0261	11.88	0.118
Soybean meal	32.1	69.1	-	0.0492	48.75	31.9	72.7	-	0.0442	42.17	0.690
Corn grain	28.5	71.2	-	0.0492	19.74	28.3	73.3	-	0.0461	23.32	0.263
Soybean hulls	22.8	76.3	-	0.0280	27.11	23.4	78.6	-	0.0262	26.12	0.544
Cottonseed meal	33.5	53.9	-	0.0300	16.88	32.2	55.3	-	0.0308	15.15	0.642
Crude Protein											
Soybean meal	14.1	88.8	-	0.0418	88.28	17.2	87.2	-	0.0406	91.08	0.445
Corn grain	51.7	49.5	-	0.0342	25.71	52.7	50.6	-	0.0314	32.44	0.190
Soybean hulls	58.6	37.5	-	0.0362	19.61	59.5	36.5	-	0.0357	24.24	0.212
Cottonseed meal	24.7	73.3	-	0.0794	32.73	25.0	73.8	-	0.0770	48.64	0.069
Neutral Detergent Fiber ^3^											
Sugarcane	-	46.9	53.1	0.0438	8.47	-	47.8	52.2	0.0448	7.58	0.656
Corn silage	-	70.4	29.6	0.0523	18.46	-	70.2	29.8	0.0535	20.05	0.365
Tifton hay	-	66.5	33.5	0.0588	12.91	-	66.4	33.6	0.0615	16.59	0.159

^1^ A, soluble fraction (g/100 g); B, insoluble and potentially degradable fraction (g/100 g); k ou λ, parameters associated with the degradation rate of the insoluble and potentially degradable fraction (h^−1^); I, undegradable fraction (g/100 g); σ^2^_Ԑ_, residual variance. ^2^ *p* value for the comparison between residual variances obtained with the complete and minimum designs. ^3^ After adjustment, the estimates of fractions B and U were adjusted according to the recommendation of Waldo et al. [47].

## Data Availability

The data generated during the current study are available from the corresponding author on reasonable request.

## Supplementary Materials

The evaluation of rumen fermentation characteristics of the animals, which is available online at https://www.mdpi.com/article/10.3390/ani12151901/s1. Refs. [30,48,49] are cited in Supplementary Materials.
