# Supplementary material for: Variability among Animals and Incubation Protocols for Ruminant In Situ Degradation Studies with Tropical Feeds
Source: Animals (Basel). 2022 Jul 26;12(15):1901. doi: 10.3390/ani12151901 (PMC9367396; doi:10.3390/ani12151901)
Supplement: Supplementary file 1 [file animals-12-01901-s001.zip › animals-1754008-supplementary.pdf]

Evaluations of ruminal environment

On d 16, rumen digesta samples were taken from the liquid-solid interface of rumen environment at 6:00, 12:00, 18:00 and 24:00 h. Samples were filtered through four layers of cheesecloth and assessed regarding pH using a potentiometer. After that, a 20-ml aliquot of ruminal fluid was combined with 5 mL of a metaphosphoric acid solution (250 g/L) and frozen (-20°C) for subsequent analysis of volatile fatty acids (VFA). Another 40-mL aliquot was combined with 1 mL of H_2SO_4 solution (9 M) and frozen (-20°C) for later analysis of ruminal ammonia-N (RAN) concentration.

The RAN concentrations were performed according the colorimetric method described by Detmann et al. (2021, method N-006/1). For VFA analysis, rumen fluid samples collected over time were pooled (2.0 mL) and centrifuged (12,000 × g for 10 min at 4°C) and supernatants were treated as described by Siegfried et al. (1984). Ruminal VFA were analyzed by HPLC (Shimadzu HPLC class VP series, model SPD 10A; Shimadzu Corporation, Kyoto, Japan) using a reverse phase column (mobile phase 0.15 M ortho-phosphoric acid) and UV detector at a wavelength of 210 nm.

On d 17, rumen digesta samples were taken from the liquid-solid interface of rumen environment at 8:00 h. Samples were filtered through four layers of cheesecloth and assessed regarding osmolarity using cryoscopy (MK 540, ITR, Esteio, RS, Brasil). On d 18, rumen digesta samples were taken three hours before feeding animals in the morning, to evaluate the specific activity of deamination (Bento et al., 2016).

The average values of different ruminal characteristics obtained along the incubation groups (Table A.1) were standardized ($\mu = 0$; $\sigma^2 = 1$) and evaluated by a

clustering procedure using the CLUSTER procedure of SAS 9.4. The clustering was based on the UPGMA method using the mean square distance as a dissimilarity measure.

The cluster analysis indicated that the five animals could be grouped into four distinct groups with respect to ruminal fermentation characteristics, as indicated by the arbitrary cut done at 60% of the maximum clustering distance (Figure S.1). Thus, the animals used in our experiment could be considered heterogeneous regarding rumen fermentation, despite of being phenotypically similar. Such a pattern adds a concrete support for all discussion presented in the main body of the article.

Table S1. Mean characterization throughout the experiment of ruminal environment characteristics of the different animals used to perform the *in situ* incubation procedures.

	Animal				
Item[1]	1	2	3	4	5
OSM	290	316	315	294	296
SAD	5.56	8.36	6.09	6.85	6.77
pH	5.71	5.50	6.17	5.20	6.10
RAN	5.67	5.07	6.39	4.51	7.87
ACET	54.6	67.6	55.0	66.8	64.5
PROP	12.3	16.3	12.5	16.5	14.4
BUT	10.2	13.3	9.8	11.9	11.1

[1] OSM, osmolarity (mOsmol/L); SAD, specific activity of deamination (nmol NH_3/mg protein per min); pH, rumen pH; RAN, rumen ammonia N (mg/dL); ACET, acetate (mmol/L); PROP, propionate (mmol/L) and BUT, butyrate (mmol/L).

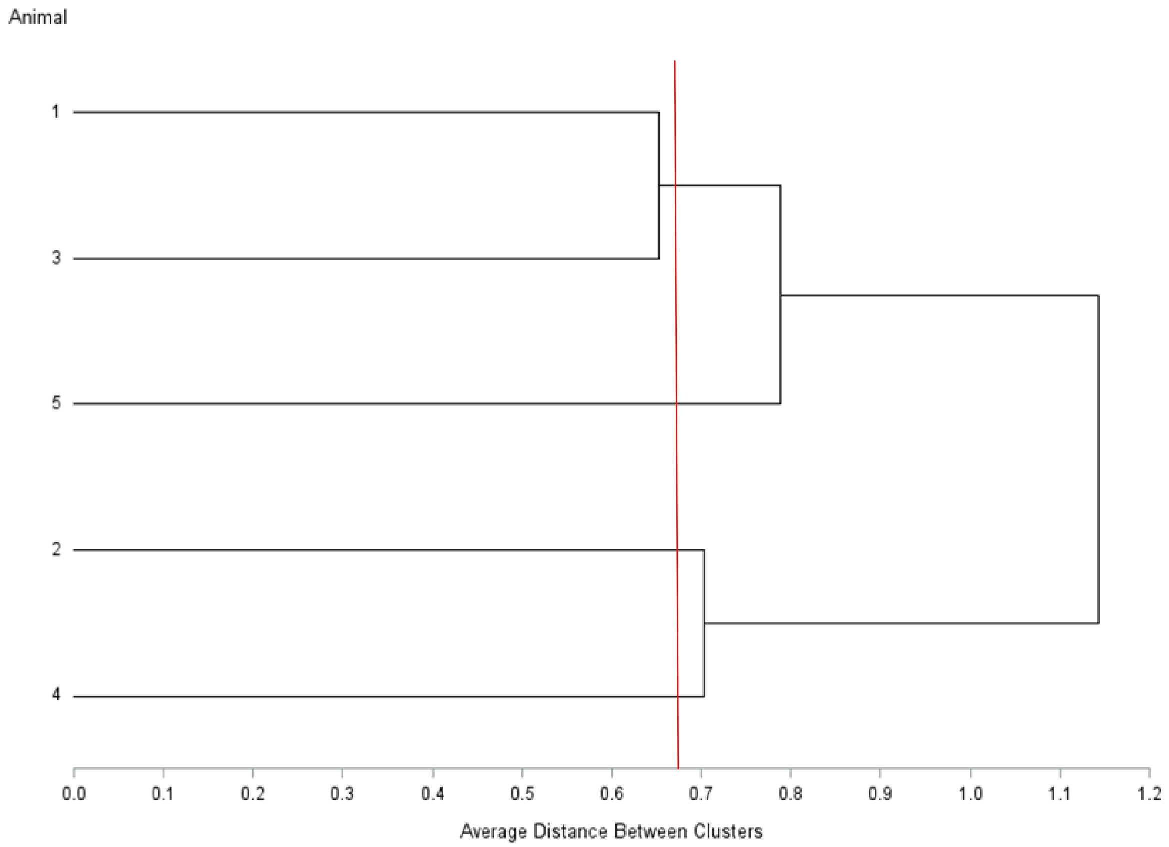

Figure S1. Cluster procedure for grouping the animals used in the *in situ* incubation procedures based on the ruminal characteristics shown in Table 4. The red line corresponds to the arbitrary cut at 60% of the maximum clustering distance (0.6 × 1.143 = 0.686).

References

Bento, C.P.B.; Azevedo, A.C.; Gomes, D.I.; Batista, E.D.; Rufino, L.M.A.; Detmann, E.; Meantovani, H.C. Effect of protein supplementation on ruminal parameters and microbial community fingerprint of Nellore steers fed tropical forages. Animal, v.10, p.44-54, 2016.

Detmann, E., Costa e Silva, L.F., Rocha, G.C., Palma, M.N.N., Rodrigues, J.P.P., 2021. Métodos para análise de alimentos. Suprema, Visconde do Rio Branco. 350p.

Siegfried, B.; Ruckermann, B.; Stumpf, R.H. Method for determination of organic acids in silage by high performance liquid chromatography. Landwirtschaftliche Forschung, v.37, p.298-304, 1984.
